# High status males invest more than high status females in lower status same-sex collaborators

**DOI:** 10.1371/journal.pone.0185408

**Published:** 2017-09-27

**Authors:** Henry Markovits, Evelyne Gauthier, Émilie Gagnon-St-Pierre, Joyce F. Benenson

**Affiliations:** 1 Département de Psychologie, Université du Québec à Montréal, Succ Centre-Ville, Montréal, Canada; 2 Faculty of Science, Emmanuel College, Boston, MA, United States of America; 3 Department of Human Evolutionary Biology, Harvard University, Cambridge, MA, United States of America; University of Reading, UNITED KINGDOM

## Abstract

Studies on human cooperation using economic games rarely include ecologically relevant factors. In studies on non-human primates however, both status and sex typically influence patterns of cooperation. Across primate species, high status individuals are more likely to cooperate, though this depends on the species-specific social structure of each sex. Based on human social structure, we predict that higher status males who interact more in hierarchical groups than females, will invest more than high status females in valued same-sex peers after successful cooperation. Across three studies, 187 male and 188 female participants cooperated with a (fictitious) same-sex partner who varied in competence. Participants then divided a reward between themselves and their partner. High status was induced in three different ways in each study, social influence, leadership and power. No overall sex difference in reward sharing was observed. Consistent with the hypothesis however, across all three studies, high status males invested more than high status females in cooperative partners, suggesting that high status males intuitively evaluate sharing rewards with same-sex partners as more beneficial.

## Introduction

Cooperation between unrelated individuals, which cannot be explained by kin selection, has generated intense interest. Utilizing computational models and economic games, researchers across evolutionary biology, mathematics, neuroscience, economics, military science, government, and psychology, have investigated the reasons individuals choose to cooperate and share rewards with unrelated individuals, rather than free-riding [1–3].

Research on non-human primates in natural and captive settings has identified several factors that influence cooperation: dominance status, kinship, sex, age, and friendship and the social structure of the species [4, 5]. Computer simulations and simple economic games of human cooperation however rarely incorporate these factors. Only sex has been extensively studied in economic games, and no consistent sex differences in cooperation have been identified [6–8]. In the following studies, we investigate the hypothesis of an interaction between sex and status, such that high-ranked males will be more generous when sharing a reward after cooperation than high-ranked females.

There is evidence from a variety of sources that status has an effect on cooperative behavior. In non-human primates, when individuals cannot obtain a reward on their own, they typically choose one other dominant individual with whom to cooperate [5]. For example, in chimpanzees (*Pan troglodytes*), one of humans’ closest living genetic relatives, community males interact in hierarchical groups. When too many females are present for the alpha to guard, cooperative mate guarding followed by sharing matings typically occurs between the alpha male and the second ranked male [9]. High-ranked males also cooperate with lower-ranked males in exchange for coalitional support in agonistic encounters or more generally to maintain their status [10–12]. Across primate species, cooperation and reward sharing most frequently occur between two individuals, and between the alpha and other higher-ranked individuals [5]. The few studies examining human behaviour using an economic game have found similar interactions [13, 14]. These have shown that only individuals who are not strong enough to win a reward on their own chose to cooperate. In addition, when given a choice, individuals chose to cooperate with a single higher status individual. Thus, both nonhuman primates and humans choose coalitional partners in order to maximize the probability of obtaining a reward.

Importantly, cooperative behavior also depends on social structure. When individuals of a species form stable groups, cooperation is more frequent and a cooperative partner’s value is higher [15]. This occurs among chimpanzee males, where many males of the community cooperate to protect their territory from or aggress against hostile neighboring communities [16] as success is based on numerical advantage [17].

Critically, among humans, there is clear evidence that sex and social structure interact. Like chimpanzee males, much research indicates that human males form large stable groups, while human females interact in unrelated dyads from an early age across a variety of cultures [18–21]. Further, within a group, males typically organize themselves hierarchically beginning in childhood [19, 21–23]. Similarly to chimpanzee males, much human male competition occurs between competing groups, which share a dominance hierarchy controlled by high status individuals [24]. This suggests that high status males should place a greater value on cooperative partners than would be expected solely by their objective value. By contrast, human females are more likely to enter into exclusive dyadic relationships [25] which are more stable when partners are of equal rank [26].

Although empirical evidence with humans is scarce, there is some evidence suggesting that high status human males are more likely than high status females to cooperate with lower status same-sex individuals. Specifically, cooperation between professors in the same department on a joint publication was found to be more common between higher and lower status men than between higher and lower status women [27]. In contrast, high status male and female professors were just as likely to cooperate with same-sex individuals of identical high status. Other studies suggest that high ranked females and males interact differently in cooperative settings, with high status male classmates interacting more equally with unrelated same-sex classmates than high status females both in early childhood [28], and in middle childhood [29]. Several studies additionally demonstrate that compared with higher-status women, higher-status men perceive lower-status same-sex peers more positively [30, 31]. However, although suggestive, a more direct measure of how much high status males and females value cooperative partners is given by reward sharing following cooperation, something that, to our knowledge, has not been directly examined.

There exist several aspects of economic behavior in which there are well-documented sex differences (see [32] for a review). There is however little direct evidence for sex differences in cooperative behavior [6, 7, 33], although see [34]. To our knowledge, status has not been included in any study of cooperative behavior despite being considered perhaps the most important factor that predicts cooperation in non-human primates [5].

The aim of the following studies was thus to examine the interaction between status and sex. In humans, high status results from a variety of sources, including positions of leadership or power [35], prestige or dominance [36], or any form of social influence. Because high status takes such differing forms, we defined status using separate definitions in each of the three following studies: social influence, leadership, and power. We specifically tested the hypothesis that higher status men would choose to invest more than higher-status women in lower-status same-sex individuals with whom they had cooperated to obtain a reward. To avoid any potential influence of prior experience, we created de novo three experimental games in which we could manipulate status and partner value in a neutral setting with anonymous partners. In each game, we modelled a situation in which a participant and a partner each make an individual, quantifiable contribution to a joint task, and as a consequence of this, receive a joint reward. The participant is then asked to distribute the reward between themselves and the partner. This basic situation is a form of dictator game with joint production, where the production phase involves obtaining a reward as a function of the combined efforts of two partners [37]. This was designed to model behavior in an organizational setting, where a task is split up into components, but rewards are distributed by a single individual. In each game, status is manipulated experimentally. Partners differed in the extent to which they contributed to the joint results, with the constraint that the relative contribution of the partner was either equal or inferior to that of the participant. This was done in order to avoid any overt conflict between high status and relative competence. These games did not examine the decision to cooperate, but looked at reward distribution following cooperation.

In our first study, we aimed to create an entirely gender-neutral context so that potential sex differences in responses to contents, talent, and attitudes towards compensation would be minimized. In this, we presented participants with a series of situations each of which showed the participant and one fictitious same-sex partner. Participants were informed on screen that the pair had succeeded in doing an unidentified joint task, and were asked to distribute a reward between themselves and the partner. Three dimensions were varied. Relative status of the participant was manipulated by varying the description of the participant as influential or not and was a between-subjects variable. For each situation, the competence level of the participant (expressed as the probability of the participant being able to finish the task alone) and that of the partner was shown. The relative difference in competence levels between participant and partner was varied as a within-subjects variable. In addition, for each situation, the reward (in points) given for successfully completing the task was shown, and was also varied as a within-subjects variable.

## Study 1

### Method-study 1

44 female and 44 male Canadian undergraduate students participated individually. After the study was explained and verbal consent obtained, the participant was seated at a computer which provided all instructions. Initially, the computer randomly assigned the participant to either the high or low status condition. In this study, status was defined as exerting influence over a group of same-sex individuals. Half of the participants of each sex were randomly assigned to the high or low status condition. In the high status condition, participants were asked to imagine that they belonged to a group in which they wielded the most influence:

Imagine that you belong to a group of same-sex students. In this group, you are the person with the most influence. Whenever you suggest an activity, the others always find your suggestions to be excellent. When you dislike someone who is not in your group, the others end up sharing your evaluation.

In the low status condition, participants were told that they belonged to a group in which they wielded the least influence:

Imagine that you belong to a group of same-sex students. In this group, you are the person with the least influence. Whenever you suggest an activity, the others always find your suggestions to be really bad. When you dislike someone who is not in your group, the others never share your evaluation.

After assignment to a status condition, participants then completed 12 trials, each of which described the results of cooperating with a fictitious partner. On each trial, the screen displayed an icon representing the participant and a fictitious partner. All icons depicted faces of the same sex as the participant. Under each icon was a number constituting the probability of being successful at completing the task on their own (i.e. their competence level), and the amount of the reward that could be gained by cooperating (expressed in terms of points). Three quantities of rewards were provided: 10, 20 or 40 points. The participant then was informed that the pair had done well enough to merit a reward. The participant then had to indicate how much of the reward to allocate to his or her partner. Note that in this experiment, points were not translated into any concrete rewards.

The participant’s probability of being successful on his or her own ranged from high (between 80 and 90%) to moderate (between 50 and 60%). This was done in order to keep a range of possible values, while insuring that participants’ competence levels were never less than partners’ competence levels. For a similar reason, partner’s probability of being successful varied from moderate (between 50 and 60%) to small (between 10 and 20%). On each trial, the partner’s competence level was either equal to, moderately less than (40 percentage points less), or much less than (70 percentage points less) the participant’s competence level. There were four combinations of relative competence: (1) Equality- participant and partner both had equally moderate competence levels, (2) Moderate superiority high- participant had a high competence level and the partner had a moderate competence level with a difference of 40, (3) Moderate superiority low- participant had moderate competence level and partner had a low competence level with a difference of 40, and (4) Large superiority- participant had a high competence level and partner had a low competence level with a difference of 70.

### Results-study 1

Preliminary analysis using a repeated measures Anova showed that amount of the reward exerted no effect, *F*(2, 83) = 1.57, *p* = n.s., and the two moderate superiority conditions yielded virtually identical behavior (*M* = .358 vs *M* = .360). Therefore, we averaged across amount of reward and across the two moderate superiority conditions. We then conducted an analysis of variance (ANOVA) with the mean proportion of reward shared with the partner as the dependent variable, relative competence of the participant (equal, moderate superiority, large inequality) as the repeated measure, and sex and status (social influence) as independent variables. This produced a significant effect of Status, *F* (1, 84) = 8.21, *p* < .01, and significant interactions between Status X Relative competence, *F* (2, 83) = 3.54, *p* < .05, and between Sex X Status, *F* (1, 84) = 4.28, *p* < .05.

**Fig 1:**
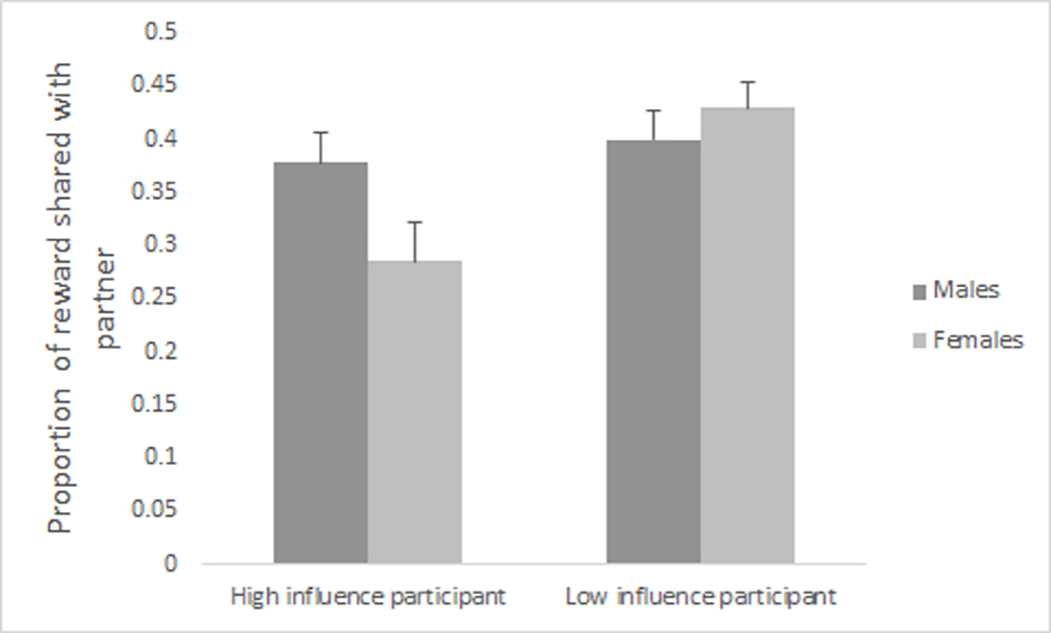
Mean (and SE) proportion of reward shared by males and females with high and low influence in Study 1. High influence males share more than high influence females with same-sex partners of all levels of competence.

Tukey’s tests, *p* < .05, demonstrated that high influence males (*M* = .38, *SE* = .03) shared a significantly larger proportion of their rewards across all relative competence levels than did high influence females (*M* = .29, *SE* = .04), see Fig 1. Low influence females (*M* = .43, *SE* = .02) and low influence males (*M* = .40 *SE* = .03) did not differ from high influence males in how much they shared, but all three groups shared significantly more with their partners than high influence females did.

Analysis of the status X relative competence interaction was done using Tukey’s tests, *p* < .05. This showed that high influence participants shared less than low influence participants when relative competence was largely unequal (High influence: *M* = .29, *SE* = .03; Low influence: *M* = .40, *SE* = .02) and when relative competence was moderately unequal (High influence: *M* = .32, *SE* = .02; Low influence: *M* = .40, *SE* = .02). No significant difference was obtained when relative competence was equal (High influence: *M* = .38, *SE* = .03; Low influence: *M* = .45, *SE* = .02). Further, among high influence participants, sharing was significantly higher with equal relative competence than with moderate unequal relative competence, which was in turn higher than with highly unequal relative competence. Among low influence participants, sharing was higher with equal relative competence than with moderately unequal relative competence, but there was no significant difference between moderately unequal and highly unequal relative competence.

Study 1 showed that after a fictitious cooperative task, high status men reported that they would invest more in fictitious partners than high status women. While interesting, these results are purely hypothetical since they rely on a scenario with no actual rewards. In Study 2 therefore, we simulated real cooperation with a partner and asked participants to distribute actual monetary rewards.

## Study 2

The goal of the second study was thus to simulate a situation in which a high status individual distributed rewards to a high or low performing partner after a cooperative activity. To this end, we modified a task created by Bediou et al. [37], in which the participant and a (fictitious, but realistic) same-sex partner separately performed an academic task, with their combined performances determining whether or not they would earn a monetary reward. Following past studies, every effort was made to make the simulation appear as realistic as possible [38], and no participant questioned its veracity. Participants were informed that partners were fictitious after the experiment was terminated.

In this study, on each round, participants completed a time-limited quiz, after which an anonymous (and fictitious) partner also completed the same quiz. Participants were informed that if the combined performance of the two exceeded a set limit, the pair would receive a monetary reward ($3), which was to be distributed by the participant. Each participant did two such rounds, for both of which the reward was given. Previous results [37] have shown that in a similar situation, reward sharing reflects the partner’s relative contribution. We thus varied this factor. On one of the rounds, the partner’s score was only 1 point less than the participant (high performing partner), while on the other the partner’s score was 1/3 of the participant’s score (low performing partner), with the order systematically inverted. Relative performance of partner was thus a within-subjects variable, while order was a between-subjects variable. Status was manipulated by inducing a leadership set. Half of the participants were told that they were the leaders due to their superior performance and were in consequence responsible for the distribution of the reward (on both rounds), while the other half were simply told to distribute the reward with no other instructions (control condition). Status was thus a between-subjects variable.

### Method-study 2

Each participant completed two computerized tasks in which a reward was contingent on both the participant and a (fictitious) anonymous same-sex partner’s performance. On one of the two tasks, the partner performed almost as well as the participant, while on the other task, the partner performed much more poorly than the participant. The order of the tasks was counterbalanced within sex.

93 female and 93 male students participated. After verbally consenting to participate, a participant was seated in front of a portable computer which displayed a set of instructions on the screen (see S1 Text for the complete set of instructions for Study 2). These stated that the participant would be working on two successive sets of problems in collaboration with two different anonymous same-sex students. These students were described as being elsewhere in the building, but would be working at the same time as the participant. On each task, if combined score of the participant and the anonymous partner were high enough, the team would be awarded a total of $3 for each trial. At the end of each problem set, after the combined results were calculated, participants were told that their team would be given the money they had earned. The participant then was given an example of the two problem types- math and verbal. Math problems involved additions, such as 257+87. Verbal problems presented scrambled letters, and participants would choose which one of a set of words that used the same letters, e.g. tfor; fort, fore). Additionally, participants were told that the anonymous peer with whom they were cooperating would also complete similar problems sets jointly with partners other than the participant. They were told that each cooperative partner would be receiving money from several other cooperative situations, and would not know how much money they had received from the participants.

**Fig 2:**
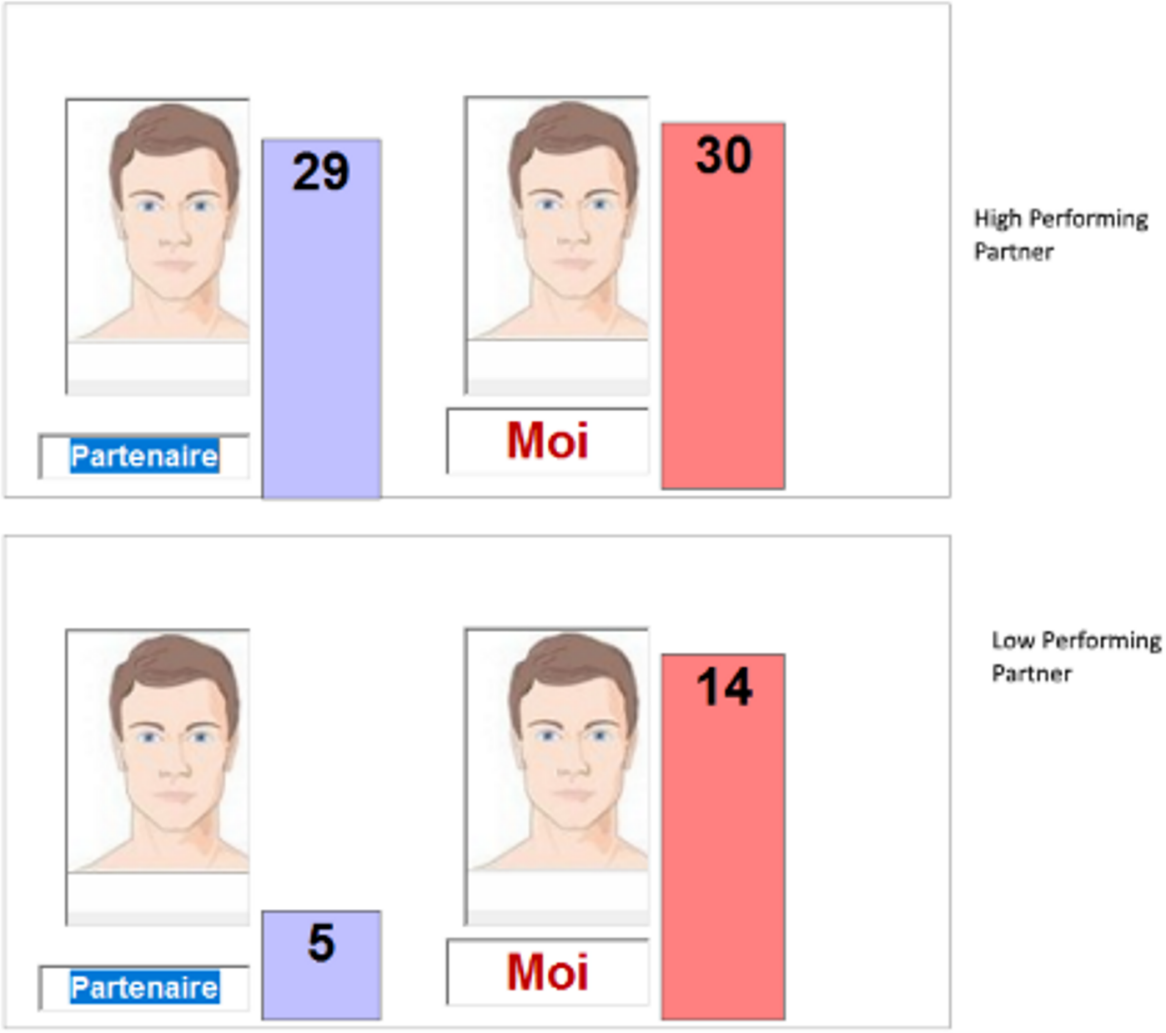
Examples of presentation of relative performance with high and low performing partners. Top of figure is a screen shot with a high performing partner (29 points), bottom is a screen shot with low performing partner (5 points)

Once the participant indicated that she or he understood the goal and the situation, the experimenter simulated a call to another experimenter seemingly to ensure that the partner would begin work on the problem set at the same time as the participant. The experimenter then left the participant to complete the problems on the computer. After the participant finished one problem set, a message appeared on the screen that the computer was contacting the network in order to find the score of the partner accompanied by a blinking signal typically used to indicate an ongoing computer process. After 30 seconds, the scores of the participant and the fictitious partner were exhibited on the screen in two bar graphs (the length of the bars were identical irrespective of the actual scores). The bar showing the participant’s score gave the real number of successful problems. High performing partners were shown as having one point less than the participant. Low performing partners were shown with about 1/3 of the participant’s scores (see Fig 2). On each of the two sets, each of which involved a different fictitious partner, participants were informed that their team had accumulated enough combined points to earn the $3.

Each computerized problem set consisting of 30 short math and verbal problems, which had been pretested for this population (mean number correct = 17). Participants were given 1 minute to do as many problems as possible. Pretesting ensured that participants really believed that they were cooperating with a same-sex individual in another room at their college [38].

Each participant was paired with two partners, one who performed almost as well as the participant (high performing) and the other poorly (low performing), with the order of the partner’s competence counterbalanced within sex. In each case, participants were told that the combined scores of the participant and their partner was sufficient to obtain the reward. After the participant viewed her or his score and that of the fictitious partner, a final set of instructions was given. 46 females and 46 males were randomly assigned to the leadership condition, with the remaining participants assigned to the control condition. In the *leadership* condition, after being shown their own and their partner’s performance, participants were told that because they had performed better, they were the team’s leader and must determine the allocation of payoffs. In the control condition, after being shown their own and their partner’s performance, participants were simply asked to allocate the payoffs. Accordingly, after each problem set, the participant had to indicate on the screen how much of the $3 they would like to keep and how much to give to their partner in increments of 10 cents. At the end of each problem set, the experimenter gave participants the money the participants indicated that they kept for themselves. Money donated to partners was put into two separate envelopes, supposedly to be distributed subsequently to the two partners.

### Results-study 2

Preliminary regression analyses determined that there was no relation between actual performance on the tasks and sharing, F(1,1) = 2.50, p = n.s. We then conducted an ANOVA with amount of money shared with the partner as the dependent variable, relative competence of the participant (high or low performing) as the repeated measure, and sex, order and status (leadership, control) as independent variables. This yielded significant main effects of partner’s performance, F (1, 178) = 7.42, p < .01, a significant two-way interaction between Partner’s performance X Order, F (1, 178) = 8.27, p < .01, and a significant 3-way interaction between Gender X Status X Partner’s performance, F (1, 178) = 8.65, p < .01. Post hoc analyses were computed using Tukey tests with *p* < .05.

Overall, participants shared significantly more money with high performing (M = 1.58, *SD* = 0.71) than low performing partners (*M* = 1.39, *SD* = 0.76), although this was modulated by order. When participants were initially paired with a high performing partner, they rewarded the low performing partner (M = 1.24, *SD* = 0.77) significantly less than the high performing partner (*M* = 1.63, *SD* = 0.82). In contrast, when participants were initially paired with a low performing partner, there was no such effect (low performing: *M* = 1.53, *SD* = 0.73 high performing: *M* = 1.53, *SD* = 0.57).

**Fig 3:**
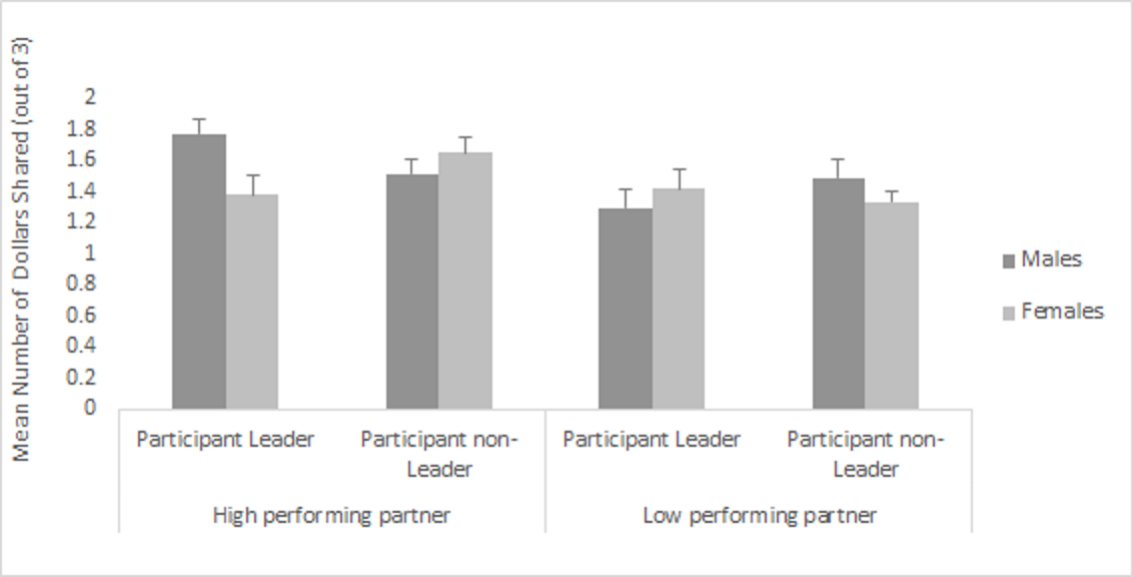
Mean (and SE) amount shared (out of a total of $3) by male and female leaders and controls with high and low performing partners in Study 2. Male leaders share more than female leaders with subordinate but high-performing same-sex partners.

Analysis of the three-way interaction showed that male leaders (*M* = 1.77, *SD* = 0.64) shared significantly more with high performing partners than female leaders did (*M* = 1.39, *SD* = 0.82), see Fig 3. In the control condition, men (*M* = 1.52, *SD* = 0.64) and women (*M* = 1.65, *SD* = 0.69) did not differ in degree of sharing with the high performing partner. No sex or status differences occurred in amount of sharing with the low performing partner.

Again, high status males again invested more than high status females but only in high-performing partners. Interestingly, the order effect showed that sharing was strongly affected by the relative difference in competence between partners. When participants started with a high performing partner, they rewarded the low performing partner less. This was not the case in the opposite order, probably because the initial reward was quite high and participants were reluctant to go beyond this level when faced with a more competent partner. The strength of this study was that participants actually enacted the role of leader, so that it was not imagined as in Study 1. As indicated in the introduction, status has differing definitions, and we thus replicated this study using a third measure of status—power.

## Study 3

### Method-study 3

51 female and 50 male students participated. After giving verbal consent, they were seated at a computer which provided instructions. Participants followed the same basic procedure as in Study 2 except that the high-performing partner was always first followed by the low-performing partner, and with a different status manipulation. Participants were placed either in the powerful or powerless condition, which was established by using previously verified implicit priming procedures. 27 females and 26 males were randomly assigned to the powerful condition, and the remainder to the powerless condition.

The basic design was identical to that used in Study 2, with the same monetary rewards, etc. The only difference was that the leadership manipulation was replaced by two priming procedures that were used to manipulate perceived power. The first procedure was used before participants engaged in the collaborative activities, and was based on the procedure devised by [39]. Participants in the powerful condition were asked to write down an episode in which they had control over other people. In the powerless condition, participants were asked to write down an episode in which they were obliged to defer to someone else. In addition, following the procedure of [40], in the powerful condition, the six words at the beginning of in the scrambled word tasks that were part of each of the two quizzes were modified to spell out high power words (these were influence, authority, wealth, chief, dominant, control; translated from the original French). In the powerless conditions, six words of the scrambled word tasks formed low power words (incapacity, obey, submission, cede, oppressed, enslaved). The high and low power words were chosen from a larger selection of words that were evaluated by an independent group of representative participants.

### Results-study 3

We then conducted an analysis of variance (ANOVA) with the amount of money shared with the partner as the dependent variable, relative competence of the participant (high or low performing) as the repeated measure, and sex and status (powerful, powerless) as independent variables. This yielded significant main effects of partner’s performance, F (1, 97) = 13.77, p < .001, and a significant two-way interaction between sex X status, F (1, 97) = 4.32, p < .05. Post hoc analysis were performed using with Tukey’s test, *p* < .05.

**Fig 4:**
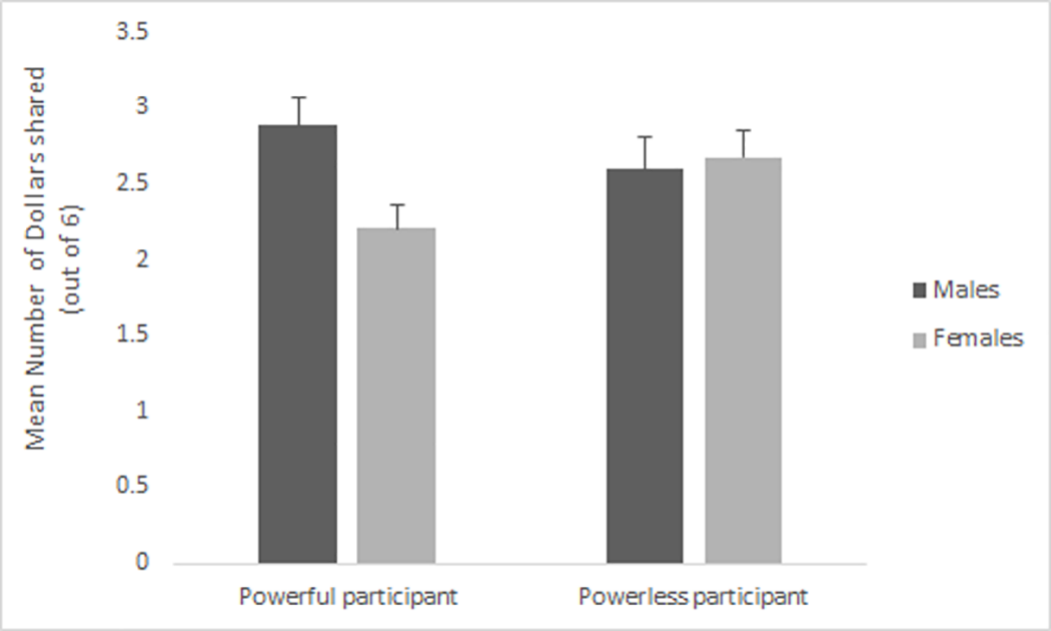
Mean (and SE) amount shared (out of a total of $6) by powerful and powerless males and females across partners in Study 3. Powerful males share more than powerful females with subordinate same-sex partners of all performance levels.

Again, participants shared significantly more with the high performing partner (M = 1.39, SD = 0.45) than the low performing partner (M = 1.19, SD = 0.63). Further, powerful males shared significantly more with high and low performing partners combined, out of a total of 6 (M = 2.59, SD = 1.04) than powerful females did (M = 2.20, SD = 0.82) as (see Fig 4).

## Discussion

Sex and status are powerful determinants of cooperative behavior in non-human primates. To our knowledge, no studies have examined the interaction between these factors. In three studies, we examined the hypothesis that high ranked males would be more invested in a cooperative partner than high ranked females. Consistent with our hypothesis, the results of all three studies show that after a successful cooperative task, high status males do indeed share more than high status females with same-sex partners. In contrast, no differences were observed between low status males and females in investment in same-sex peers. Further, in Studies 1 and 3 high status males invested more than high status females in both high and low performing subordinates, although in Study 2, this difference was found only with higher performing partners. The effect occurred using three different instantiations of high status. In Study 1, participants were asked to imagine that they were the most influential member of a group; in Study 2 participants were told that they were the leader due to their higher performance on the joint task and in Study 3, they were given two different forms of power prime. In both Studies 2 and 3, participants shared monetary rewards with their partners, believing that their success was due to their joint efforts. Results are consistent with the interpretation that during a cooperative venture, high status human males are more predisposed than high status females to value the maintenance of a group and thus are willing to donate more of a joint reward to a partner.

It is useful to put these results into a wider context. Although status is a complex idea, one underlying component is priority of access to resources [41]. This would lead to the simple expectation that in most situations involving sharing of rewards, high status individuals would tend to retain a larger share. This is generally consistent with the behavior of the high status females in these studies. The behavior of the high status males thus appears to be somewhat anomalous, since they show a stronger tendency to a more altruistic level of sharing than their rank would suggest. Our hypothesis here is that high status males are using increased altruistic sharing as a way to increase the social bonding of lower status individuals to an (intuitive) group. The fact that sharing was somewhat more consistent with lower status and higher performing partners than with lower status and lower performing partners (although this was only found in Study 2), reinforces this idea, since high performing partners would be relatively higher value group members. Such a mechanism is similar to the notion of sharing-to-enhance-status proposed by Brosnan and de Waal [42], which suggests that altruistic sharing can sometimes be explained by associated benefits to the sharer, despite the loss of resources. Although the exact proximal mechanism remains an open question, our results do show that high status males are more willing to sacrifice some of the advantages of high status in order to reward a cooperative partner, more so than high status females.

It is also useful to note that in all three studies, sharing was generally greater when putative partners contributed more to the cooperative activity (although this was modulated by the order effect found in Study 2). These results were consistent with those of other studies using a different paradigm [34], and indicate that our participants were both attentive to the details of the information provided, and were making rational decisions. It is also interesting to note that there were clear differences in the extent to which participants donated more than half of the total reward. In Studies 2 and 3 which used real money, the percentage of participants doing so was 16.7% and 6.0% respectively. Since these tasks varied mostly by the way that status was instantiated, this difference was probably due to this factor, although which aspects were involved remains an open question.

These results have some practical implications. Cooperative activities are at the heart of modern economic activity. Most companies also have clear hierarchical structures, with institutionalized status markers. Our results show that high status males are more willing to forgo some of the advantages of status in order to reward collaborators, which would have the associated effect of increasing the bonding of the latter to the group led by the high status male. Coupled with the increased tendency of high status males to actually cooperate with lower status peers [26], this implies that males have some clear intuitions that lead to an emphasis on group interactions, something that is particularly important for advancement within a modern corporate structure.

The current results conform closely to the theoretical prediction that the structural properties of human females’ and males’ relationships facilitate different types of investment. If this interpretation is correct, then promoting group identity, and reducing the intense focus on one individual at a time, could encourage high-ranked females to invest more heavily in their lower-ranked peers. Simply raising high status girls’ and women’s awareness of potentially intuitive reactions to low status same-sex individuals also could exert a strong impact on young girls’ and women’s advancement across contexts. Future research is necessary using objective measures of status, competence, and level of investment in other contexts, such as business, the military, government, and educational and religious organizations, to validate and permit generalization of the results.

## Ethics

The studies described in this article have received ethical approval from the IRB of the Université du Québec à Montréal.

## Supporting information

S1 Dataset Data, study 1. (XLS)

S2 Dataset Data, study 2. (XLS)

S3 Dataset Data, study 3. (XLS)

S1 Text Instructions study 2. (DOCX)
