# Supplementary material for: High status males invest more than high status females in lower status same-sex collaborators
Source: PLoS One. 2017 Sep 27;12(9):e0185408. doi: 10.1371/journal.pone.0185408 (PMC5617204; doi:10.1371/journal.pone.0185408)
Supplement: S1 Text — (DOCX) [file pone.0185408.s004.docx]

Complete instruction set for Study 2, experimental condition

“You will work together with another girl (boy) who is a student at your instituion, but who will be working in another room. You and your partner will each be given one minute to answer a series of problems. For each problem, you must find the right answer as fast as possible. Each problem is worth between 1 and 3 points (depending on its difficulty). There are two types of problem (1) verbal or (2) mathematical. For the verbal problems, you will see a set of letters and you must choose the word that has the same letters among the three options. For example, you see EUBL and you have a choice between BLUE, BEOT and RULE, you must choose BLUE. The mathematical problems are additions, such as 4+17. You will be given a maximum of 20 problems. There will be a coloured bar that will indicate how much time you have left. Click CONTINUE for further instructions.”

“At the end, you will be given the number of points that correspond to your performance. At the same time, your partner will be doing the same problems. When they have finished, you will see how many points they have accumulated. If you and your partner obtain enough combined points between you, your team will win some money. The person that has obtained the most individual points will be the leader of the team and will determine how any money won will be distributed. Click CONTINUE for further instructions.”

“Now, you will received a series of problems. Answer as quickely as you can when you are certain of your response. You do not have to answer all the problems, you will get more points by being certain of your answers. Attention, if you see letters, you must choose the word with the same letters. If you see an addition, you must choose the result of the addition. Before continuing, call the experimenter who will determine who your partner will be. Attention, when the button below is clicked, the problem series will start immediately.”

“You can see above how many points you have accumulated. Click on the CONTINUE button to see what the performance of your partner is (this takes some time, since it is communcated via a network).”

“Your team has received enough points to win the amount shown above. Since you have obtained more points than your partner, you are the leader of the team. As leader, you must determine how to split up the money between yourself and your partner, will you will do by using the scroll bar. Test this out to see how it works.”

“Now you must make the final split of the money won. Your choice will be completely anonymous. Your partner will play with other players. The amount of money that you give them will be added onto whatever is received from the other players, and they will never know how much you have given. You will receive your personal total at the end of the experiment.”

“Now, you will repeat this game another time. This time, you will work with a different partner, who is also a student at your institution. Before continuing, call the experimenter who will determine who your partner will be. Attention, when the button below is clicked, the problem series will start immediately.”
